# Prognostic significance of stem cell-related marker expression and its correlation with histologic subtypes in lung adenocarcinoma

**DOI:** 10.18632/oncotarget.9894

**Published:** 2016-06-07

**Authors:** Eunhyang Park, Soo Young Park, Ping-Li Sun, Yan Jin, Ji Eun Kim, Sanghoon Jheon, Kwhanmien Kim, Choon Taek Lee, Hyojin Kim, Jin-Haeng Chung

**Affiliations:** ^1^ Department of Pathology, Seoul National University Hospital, Seoul, Republic of Korea; ^2^ Department of Pathology, Seoul National University Bundang Hospital, Seongnam, Republic of Korea; ^3^ Department of Pathology, Seoul National University Boramae Hospital, Seoul, Republic of Korea; ^4^ Department of Thoracic and Cardiovascular Surgery, Seoul National University Bundang Hospital, Seongnam, Republic of Korea; ^5^ Department of Internal Medicine, Seoul National University Bundang Hospital, Seongnam, Republic of Korea; ^6^ Department of Pathology, Jilin University Second Hospital, Changchun, China; ^7^ Department of Pathology, Fudan University Shanghai Cancer Center, Shanghai, China

**Keywords:** cancer stem cell marker, immunohistochemistry, lung cancer, adenocarcinoma, Nanog

## Abstract

Cancer stem cells (CSCs) are a small subset of tumor cells that exhibit stem cell-like properties and contribute in treatment failure. To clarify the expression and prognostic significance of several CSC markers in non-small cell lung cancer, we retrospectively analyzed 368 patients with adenocarcinoma (*n* = 226) or squamous cell carcinoma (*n* = 142). We correlated the expression of six CSC markers – CD133, CD44, aldehyde dehydrogenase 1 (ALDH1), sex determining region Y-box 2 (SOX2), octamer binding transcription factor 4 (OCT4), and Nanog – with clinicopathologic and molecular variables and survival outcomes. In adenocarcinoma, CD133, ALDH1 and CD44 expression was associated with low pathologic stage and absence of lymphovascular invasion, while Nanog expression correlated with high histologic grade, lymphatic invasion and increased expression of Snail-1, a transcription factor associated with epithelial-mesenchymal transition. CSC marker expression was also associated with histologic subtypes in adenocarcinoma. Multivariate analysis showed that high Nanog expression was an independent factor associated with a poor prognosis in adenocarcinoma. CSC markers had no prognostic value in squamous cell carcinoma. These results suggest that Nanog is an independent negative prognostic factor that may be associated with epithelial-mesenchymal transition in lung adenocarcinoma.

## INTRODUCTION

The overall prognosis of lung cancer is poor. This is largely due to its often late presentation, high recurrence frequency and lack of curative systemic therapy. According to the recently proposed cancer stem cell (CSC) theory, cancers are maintained by subpopulations of tumor cells that possess stem or progenitor cell-like characteristics. These cells exhibit pluripotency and self-renewal properties, and give rise to a heterogeneous population of tumor cells [1–3]. CSCs also appear to have lower proliferation rates and higher expression of DNA repair and anti-apoptotic genes than normal cells, which can contribute in treatment failure [4, 5].

CSCs can be distinguished from other cancer cells on the basis of specific markers. In non-small cell lung cancer (NSCLC) cells, for example, CD133 and CD44 are CSC markers that confer drug resistance and stem cell-like properties [6–9]. Aldehyde dehydrogenase 1 (ALDH1), a detoxification enzyme, is also a marker for human lung CSCs [10]. In addition, the embryonic stem cell (ESC) transcription factors sex determining region Y-box 2 (SOX2), octamer binding transcription factor 4 (OCT4) and Nanog all share the biological properties of CSCs and promote tumorigenesis [11–14]. Although the clinical impact of these markers is unclear, they may have a prognostic or predictive value in NSCLC.

During epithelial mesenchymal transition (EMT), epithelial cells lose their normal properties, such as cell-cell adhesion and E-cadherin expression, and acquire the characteristics of stem or tumor cells [15, 16]. Notably, maintenance of the stem cell state depends on both EMT-inducing and stem cell maintenance signals [15]. In NSCLC, however, little is known about the underlying mechanisms or clinical significance of these processes.

To address these gaps in our knowledge, we investigated three aspects of CSC markers in NSCLC. We first examined the expression pattern of six putative CSC markers and their clinicopathologic and prognostic significance. We then assessed the expression of these markers in different histologic and molecular subtypes of adenocarcinoma (ADC). Finally, we tested whether there is a correlation between the expression of CSC and EMT markers.

## RESULTS

### Patient characteristics

The clinicopathologic characteristics of the patients in this study are summarized in Supplementary Table S1. Briefly, there were 247 men (67.1%) and 121 women (32.9%) with a median age of 63.9 years (range: 21–83 years). The majority of patients had a smoking history (*n* = 224; 60.9%). Squamous cell carcinoma (SqCC) patients were more likely to be male, older, and have a history of smoking than ADC patients. The pathologic stage was I in 156 (42.4%) patients, II in 95 (25.8%) patients, III in 105 (28.5%) patients, and IV in 12 (3.3%) patients.

### Expression of CSC markers in healthy and cancerous lung tissue

**Figure 1:**
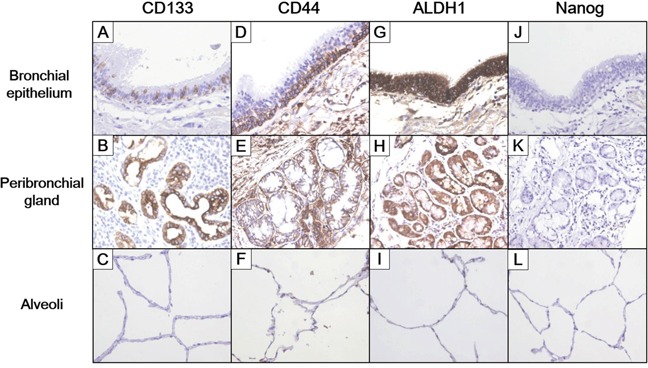
Expression pattern of cancer stem cell markers in normal lung tissue Levels of CD133 **(A-C)**, CD44 **(D-F)**, ALDH1 **(G-I)** and Nanog **(J-L)** expression were determined in healthy bronchial epithelium, peribronchial glands and alveoli (20x magnification).

In healthy lung tissue, CD133 was strongly expressed in peribronchial mucus glands and scattered throughout the bronchial epithelium, but it was not detected in type II pneumocytes. CD44 was expressed in peribronchial mucus glands and basal cells of the bronchial epithelium, but not in completely differentiated epithelial cells, such as bronchial columnar cells and type II pneumocytes. ALDH1 was expressed in all layers of the bronchial epithelium and in peribronchial mucus glands, but not in type II pneumocytes. SOX2, OCT4 and Nanog were not expressed in healthy lung tissue (Figure 1).

**Figure 2:**
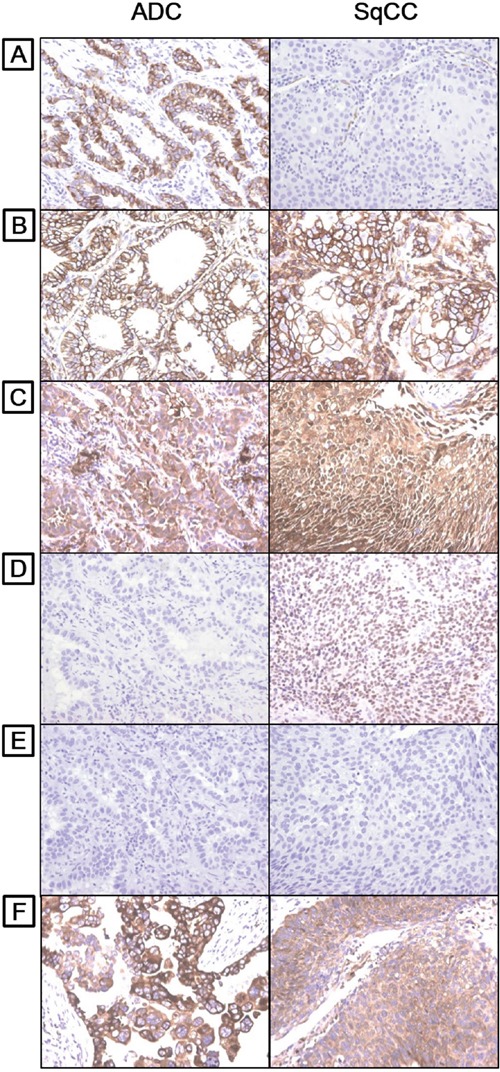
Distinct expression patterns of CSC markers in lung adenocarcinoma and squamous cell carcinoma **A.** CD133, **B.** CD44, **C.** ALDH1, **D.** SOX2, **E.** OCT4, and **F.** Nanog (10x magnification).

CSC markers showed diffuse homogenous expression in lung cancer tissue. Within cancer cells, CD133 and CD44 were detected in the membrane and cytoplasm, while ALDH1 and Nanog were found only in the cytoplasm. The transcription factors SOX2 and OCT4 were found exclusively in the nucleus (Figure 2).

### Correlations between CSC marker expression and the clinicopathologic features of lung ADC and SqCC

As shown in Figure 2, CSC markers were differently expressed in lung ADC and SqCC. In ADC, CD133, CD44, ALDH1 and Nanog were expressed in 46.9%, 33.6%, 47.3%, and 42.5% of tumor cells, respectively (Table 1). CD133 expression correlated significantly with small tumor diameter (≤3 cm; *p* < 0.001), absence of pleural and vascular invasion (*p* = 0.011 and 0.004, respectively) and low pathologic stage (*p* = 0.008). Expression of CD44 and ALDH1 correlated significantly with absence of lymphatic invasion (*p* = 0.041 and <0.001, respectively) and low pathologic stage (*p* = 0.024 and 0.037, respectively). By contrast, Nanog expression correlated significantly with large tumor diameter (>3 cm; *p =* 0.01) and lymphatic invasion (*p* = 0.01) (Table 1). SOX2 and OCT4 were rarely expressed in ADC (1.3% and 0.8%, respectively).

**Table 1:** Correlations between cancer stem cell marker expression and clinicopathologic characteristics in lung adenocarcinoma

	Markers, *n* (%)												
	CD133			CD44			ALDH1			Nanog		
	Exp	No	*p*	Exp	No	*p*	Exp	No	*p*	Exp	No	*p*
**Tumor size**												
**≤3 cm**	78 (73.6)	51 (42.5)	<0.001^*^	51 (67.1)	78 (52.0)	0.033^*^	63 (58.9)	66 (55.5)	>0.05	45 (46.9)	84 (64.6)	0.01^*^
**>3 cm**	28 (26.4)	69 (57.5)		25 (32.9)	72 (48.0)		44 (41.1)	53 (44.5)		51 (53.1)	46 (35.4)	
**Pleural invasion**												
**Absent**	66 (62.3)	54 (45.0)	0.011^*^	42 (55.3)	78 (52.0)	>0.05	61 (57.0)	59 (49.6)	>0.05	45 (46.9)	75 (57.7)	>0.05
**Present**	40 (37.7)	66 (55.0)		34 (44.7)	72 (48.0)		46 (43.0)	60 (50.4)		51 (53.1)	55 (42.3)	
**Vascular invasion**												
**Absent**	100 (94.3)	98 (81.7)	0.004^*^	67 (88.2)	131 (87.3)	>0.05	98 (91.6)	100 (84.0)	>0.05	85 (88.5)	113 (86.9)	>0.05
**Present**	6 (5.7)	22 (18.3)		9 (11.8)	19 (12.7)		9 (8.4)	19 (16.0)		11 (11.5)	17 (13.1)	
**Lymphatic invasion**												
**Absent**	61 (57.5)	59 (49.2)	>0.05	47 (61.8)	73 (48.7)	0.041^*^	70 (65.4)	50 (42.0)	<0.001^*^	41 (42.7)	79 (60.8)	0.01^*^
**Present**	45 (42.5)	61 (50.8)		29 (38.2)	77 (51.3)		37 (34.6)	69 (58.0)		55 (57.3)	51 (39.2)	
**Pathologic stage**												
**I**	63 (59.4)	48 (40.0)	0.008^*^	48 (63.2)	63 (42.0)	0.024^*^	61 (57.0)	50 (42.0)	0.037^*^	44 (45.8)	67 (51.5)	>0.05
**II**	16 (15.1)	20 (16.7)		8 (10.5)	28 (18.7)		19 (17.8)	17 (14.3)		14 (14.6)	22 (16.9)	
**III**	26 (24.5)	44 (36.7)		17 (22.4)	53 (35.3)		24 (22.4)	46 (38.7)		33 (34.3)	37 (28.5)	
**IV**	1 (1.0)	8 (6.7)		3 (3.9)	6 (4.0)		3 (2.8)	6 (5.0)		5 (5.2)	4 (3.1)	
**E-cadherin**												
**Loss**	15 (14.2)	39 (32.5)	0.002^*^	16 (21.1)	38 (25.3)	>0.05	22 (20.6)	32 (26.9)	>0.05	23 (24.0)	31 (23.8)	>0.05
**No loss**	91 (85.8)	81 (67.5)		60 (78.9)	112 (74.7)		85 (79.4)	87 (73.1)		73 (76.0)	99 (76.2)	
**Snail1**												
**Positive**	52 (49.0)	56 (46.7)	>0.05	31 (40.8)	77 (51.3)	>0.05	47 (43.9)	61 (51.2)	>0.05	55 (57.3)	53 (40.8)	0.032^*^
**Negative**	54 (51.0)	64 (53.3)		45 (59.2)	73 (48.7)		60 (56.1)	58 (48.8)		41 (42.7)	77 (59.2)	
**Total**	**106 (46.9)**	**120 (53.1)**		**76 (33.6)**	**150 (66.4)**		**107 (47.3)**	**119 (52.6)**		**96 (42.5)**	**130 (57.5)**	

Abbreviations: CD, cluster of differentiation; ALDH1, aldehyde dehydrogenase 1; Exp, expression

* Statistically significant (*p* < 0.05)

In SqCC, CD44, ALDH1, SOX2 and Nanog were expressed in 88.0%, 73.2%, 71.1%, and 92.2% of tumor cells, respectively. No CD133 or OCT4 expression was detected in SqCC. There was no significant association between CSC marker expression and clinicopathologic variables in SqCC (Supplementary Table S2).

Expression of some CSC markers also significantly correlated with EMT marker expression (Table 1). CD133 expression correlated significantly with E-cadherin expression (*p* = 0.002). Nanog expression was not associated with E-cadherin expression, but significantly correlated with increased Snail-1 expression (*p* = 0.032).

### Correlations between CSC marker expression and histologic subtypes of lung ADC

**Figure 3:**
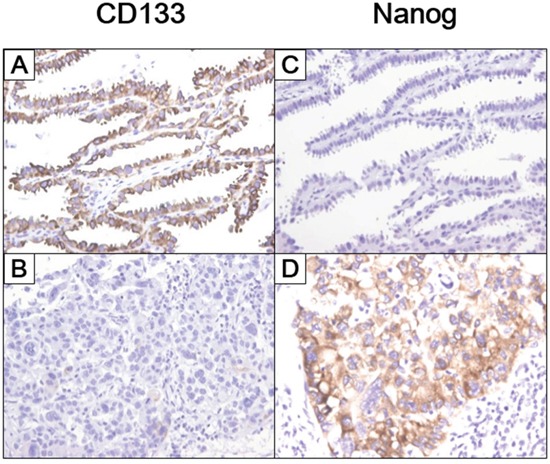
CSC marker expression in histologic subtypes of lung adenocarcinoma **A-B.** CD133 was more frequently expressed in lepidic components and less frequently expressed in solid components. **C-D.** Nanog was less frequently expressed in lepidic components and more frequently expressed in solid components (10x magnification).

Correlations between the expression of four CSC markers and histologic subtypes in the 226 ADC patients are shown in Table 2. CD133, CD44, and ALDH1 showed a trend toward expression in grade 1 ADCs, and CD44 expression significantly correlated with low histologic grade (*p* = 0.003). By contrast, Nanog expression significantly correlated with high histologic grade (*p* = 0.001). In addition, CD133 and CD44 expression significantly correlated with predominantly lepidic subtypes (*p* = 0.039 and 0.027, respectively), whereas Nanog expression correlated with predominantly solid subtypes (*p* < 0.001). CD133 was expressed significantly more frequently when the lepidic subtype was present (*p* = 0.001) and less frequently the when the solid subtype was present (*p* = 0.018) (Figure 3A and 3B). Nanog was expressed significantly more frequently when the solid subtype was present (*p* < 0.001) (Figure 3C and 3D).

**Table 2:** Correlations between cancer stem cell marker expression and histologic subtypes of lung adenocarcinoma

	Markers, *n* (%)												
	Total	CD133			CD44			ALDH1			Nanog		
	*n*	Exp	No	*p*	Exp	No	*p*	Exp	No	*p*	Exp	No	*p*
**Histologic grade**^†^													
**Grade 1**	23	13 (56.5)	10 (43.5)	>0.05	15 (65.2)	8 (34.8)	0.003^*^	15 (65.2)	8 (34.8)	0.06	5 (21.7)	18 (78.3)	0.001^*^
**Grade 2**	156	76 (48.7)	80 (51.3)		45 (28.8)	111 (71.2)		75 (48.1)	81 (51.9)		61 (39.1)	95 (60.9)	
**Grade 3**	47	17 (36.2)	30 (63.8)		16 (34.0)	31 (66.0)		17 (36.2)	30 (63.8)		30 (63.8)	17 (36.2)	
**Predominant subtype**													
**Lepidic**	13	7 (53.8)	6 (46.2)	0.039^*^	9 (69.2)	4 (30.8)	0.027^*^	10 (76.9)	3 (23.1)	>0.05	1 (7.7)	12 (92.3)	<0.001^*^
**Acinar**	154	77 (50.0)	77 (50.0)		51 (33.1)	103 (66.9)		71 (46.1)	83 (53.9)		62 (40.3)	92 (59.7)	
**Papillary**	24	14 (58.3)	10 (41.7)		4 (16.7)	20 (83.3)		9 (37.5)	15 (62.5)		10 (41.7)	14 (58.3)	
**Solid**	30	6 (20.0)	24 (80.0)		10 (33.3)	20 (66.7)		13 (43.3)	17 (56.7)		23 (76.7)	7 (23.3)	
**Micropapillary**	1	0	1 (100)		1 (100)	0		1 (100)	0		0	1 (100)	
**Invasive mucinous**	4	2 (50.0)	2 (50.0)		1 (25.0)	3 (75.0)		3 (75.0)	1 (25.0)		0	4 (100)	
**Lepidic subtype**													
**Absent**	147	57 (38.8)	90 (61.2)	0.001^*^	48 (32.7)	99 (67.3)	>0.05	64 (43.5)	83 (56.5)	>0.05	69 (46.9)	78 (53.1)	0.06
**Present**	79	49 (62.0)	30 (38.0)		28 (35.4)	51 (64.6)		43 (54.4)	36 (45.6)		27 (34.2)	52 (65.8)	
**Solid subtype**													
**Absent**	173	89 (51.4)	84 (48.6)	0.018^*^	58 (33.5)	115 (66.5)	>0.05	86 (49.7)	87 (50.3)	>0.05	60 (34.7)	113 (65.3)	<0.001^*^
**Present**	53	17 (32.1)	36 (67.9)		18 (34.0)	35 (66.0)		21 (39.6)	32 (60.4)		36 (67.9)	17 (32.1)	

Abbreviations: CD, cluster of differentiation; ALDH1, aldehyde dehydrogenase 1; Exp, expression

† grade 1, lepidic; grade 2, acinar and papillary; grade 3, micropapillary and solid

* Statistically significant (*p* < 0.05)

### Correlations between CSC marker expression and the molecular subtypes of lung ADC

Epidermal growth factor receptor gene (*EGFR*) and *KRAS* mutations and *ALK* rearrangement were present in 47.9%, 8.2% and 8.6% of the ADC patients, respectively. In three patients, both *EGFR* and *KRAS* mutations were detected. *ALK* rearrangement was not observed in patients with *EGFR* or *KRAS* mutations. *EGFR* and *KRAS* mutations and *ALK* rearrangement showed no association with the expression of CSC markers (data not shown).

### Survival analyses

**Figure 4:**
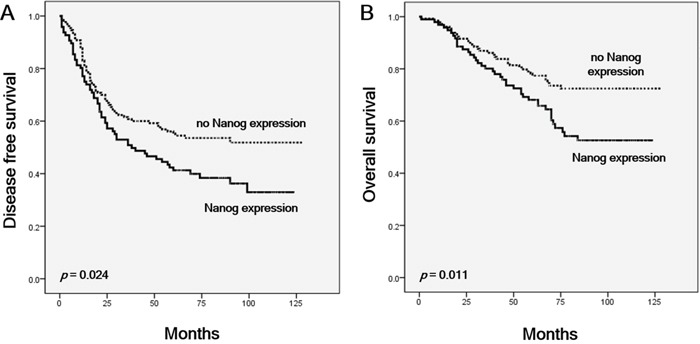
Kaplan-Meier curves showing survival among patients with and without Nanog expression in lung adenocarcinoma **A.** Disease-free survival. **B.** Overall survival. Differences were evaluated using the log-rank test.

In lung ADC patients, univariate analysis showed CD133 and ALDH1 expression to be significantly associated with better disease-free survival (DFS) and overall survival (OS). Pleural invasion, vascular invasion, lymphatic invasion, advanced pathologic stage and Nanog expression were all significantly associated with poor DFS and OS (Table 3, Figure 4). Multivariate analysis revealed that Nanog expression, vascular invasion, and pathologic stage were independent prognostic factors for poor DFS and OS (Table 3). CSC markers did not have significant prognostic value in SqCC patients (Supplementary Figure S1).

**Table 3:** Univariate and multivariate analyses of factors associated with disease-free and overall survival in lung adenocarcinoma patients

Factor	Category	Disease-free survival			Overall survival		
		Univariate	Multivariate		Univariate	Multivariate	
		*p*	*p*	HR (95% CI)	*p*	*p*	HR (95% CI)
Pleural invasion	Present vs. Absent	<0.001^*^	0.079	1.414 (0.961–2.082)	<0.001^*^	0.222	1.358 (0.831–2.219)
Vascular invasion	Present vs. Absent	<0.001^*^	<0.001^*^	2.533 (1.535–4.180)	<0.001^*^	0.037^*^	1.858 (1.037–3.327)
Lymphatic invasion	Present vs. Absent	<0.001^*^	0.318	1.240 (0.813–1.891)	<0.001^*^	0.588	1.169 (0.664–2.059)
Pathologic stage	IV vs. I, II, III	<0.001^*^	<0.001^*^	4.782 (1.965–11.637)	<0.001^*^	<0.001^*^	8.445 (2.900–24.596)
CD133 expression	Present vs. Absent	0.018^*^	0.939	1.105 (0.688–1.498)	0.007^*^	0.27	0.757 (0.462–1.241)
CD44 expression	Present vs. Absent	0.104	NA	NA	0.147	NA	NA
ALDH1 expression	Present vs. Absent	0.018^*^	0.112	0.735 (0.502–1.075)	0.042^*^	0.505	0.847 (0.519–1.381)
Nanog expression	Present vs. Absent	0.024^*^	0.03^*^	1.541 (1.043–2.278)	0.011^*^	0.032^*^	1.700 (1.048–2.760)

Abbreviations: HR, hazard ratio; CI, confidence interval; CD, cluster of differentiation; ALDH1, aldehyde dehydrogenase 1; NA, not available

* Statistically significant (p < 0.05)

## DISCUSSION

The goal of this study was to investigate the clinicopathologic and prognostic significance of CSC marker expression in histologic subtypes of ADC and SqCC. Our results showed that CSC markers have distinct expression profiles in the two cancers. Although several studies have reported on the clinicopathologic implications of CSC markers in NSCLC, there is little data comparing CSC marker expression in ADC and SqCC. In SqCC, we found no correlation between expression of CSC markers and any clinicopathologic variable or survival outcome. This result is in line with an earlier report that a higher percentage of survival-related and poor-differentiation-related genes were expressed in the CSC gene set in lung ADC than in SqCC [17]. Our results suggest that CSC markers do not correlate with any known genes or pathways that control tumor differentiation or affect survival in SqCC.

We next examined the association between CSC marker expression and prognostic outcomes in lung ADC. In previous studies, CD133 expression correlated with poor prognosis or was not associated with prognostic outcomes in lung cancer. However, our data indicate that CD133-positive tumors are indolent and significantly associated with better outcomes than CD133-negative tumors [18–21]. Moreover, CD44 expression correlated with less aggressive tumor behavior, though the association with survival was not statistically significant. The association of CD133 and CD44 expression with longer survival in ADC would appear counterintuitive, as tumors enriched in stem cell properties are expected to be more aggressive. One possible explanation with respect to CD133 is that the contribution of CD133 to cell-cell adhesion and its association with E-cadherin expression reduces tumor aggressiveness [22]. Moreover, CD133 expression is influenced by complex epigenetic, tissue-specific, spatiotemporal and tumor microenvironmental factors. Consequently, not all tumor-initiating cells express CD133, nor are all CD133-positive cells tumorigenic [23]. With respect to CD44, the interaction between CD44 and its ligand, hyaluronic acid, may inhibit angiogenesis and tumor progression [24]. The current evidence thus suggests that CD133 and CD44 should not be considered general CSC markers in NSCLC. Further study is needed to clarify the biological functions and clinical applications of these markers in NSCLC.

In contrast to CD133 and CD44, the prognostic significance of ALDH1 expression in lung ADC is largely unknown. Results from several studies in other tumors suggest ALDH1 is more likely to be expressed in a tumor if it is also present in the corresponding healthy tissue, and that loss of ALDH1 expression may be a step in the carcinogenetic process [25]. In our study, ALDH1 was expressed in all layers of the bronchial epithelium and was associated with low pathologic stage and better survival outcomes in lung ADC, which is consistent with the findings of Dimou et al. [26].

There have been few studies on the prognostic significance of Nanog expression in lung cancer [27]. Our results show that in lung ADC, Nanog expression is associated with not only a poor prognosis but also aggressive pathologic features such as poor tumor differentiation and a solid growth pattern. These findings are consistent with those of Meng et al. [28], who suggested Nanog is an important contributor to EMT that enhances tumor cell proliferation, invasion and motility. In addition, we also showed that Nanog expression correlates with Snail-1 expression, which is an EMT-inducing transcription factor. Taken together, these findings suggest Nanog expression is an independent prognostic factor for poor survival in lung ADC patients, and may therefore be a novel therapeutic target.

We also found that CSC marker expression was significantly associated with histologic subtype. Levels of CD133, CD44 and ALDH1 expression were higher in the lepidic subtype of ADC, while Nanog was highly expressed in the solid subtype. Lung ADC is a histologically heterogeneous subset of NSCLC, and survival outcomes differ depending on the histologic subtype across all pathologic stages [29, 30]. Our results suggest that CSC markers may reflect the differentiation state of lung cancers and contribute to their phenotypic heterogeneity. In addition, the association of CSC markers with histologic subtypes suggests that comprehensive histologic subtyping following the IASLC/ATS/ERS classification may provide additional insight into the genesis of lung ADC within the context of CSC theory.

In conclusion, we have shown that CSC markers may be prognostic factors in NSCLC, and high Nanog expression is an independent prognostic factor for poor survival that may be associated with EMT features in ADC patients. In addition, the clinicopathologic implication of CSC markers in lung ADC differed from those in tumors arising from other organs. Thus, the impact of CSC marker expression should be considered in a tumor/organ specific manner.

## MATERIALS AND METHODS

### Patients and data collection

We retrospectively enrolled 368 NSCLC patients with ADC (*n* = 226) or SqCC (*n* = 142) who had undergone surgical resection at Seoul National University Bundang Hospital between May 2003 and December 2008. None of these patients received preoperative chemotherapy or radiation therapy. Clinicopathologic data was obtained from the medical records and pathology reports. The pathological stage of the tumors was determined according to the guidelines in the Cancer Staging Manual of the American Joint Committee on Cancer (7^th^ edition) [31]. The study protocol was approved by the Institutional Review Board of Seoul National University Bundang Hospital.

### Histological analyses

All resected tumor specimens were fixed with formalin and then stained with hematoxylin and eosin (H&E). All H&E slides were carefully reviewed by two of the authors (E.P. and H.K.) to determine tumor subtype. ADC *in situ* and minimally invasive ADC samples were excluded from the study. All other invasive ADC samples were categorized as lepidic, papillary, acinar, micropapillary, solid, or invasive mucinous according to the International Association for the Study of Lung Cancer/American Thoracic Society/European Respiratory Society (IASLC/ATS/ERS) classification of lung cancer [32]. These histologic subtypes were used to determine tumor grade (lepidic, grade 1; acinar and papillary, grade 2; and micropapillary and solid, grade 3).

### Construction of tissue microarrays

Tissue microarray (TMA) blocks were constructed from the most representative areas of paraffin blocks by Superbiochips Laboratories (Seoul, Korea), as previously described [33].

### Immunohistochemical analysis

Immunohistochemistry (IHC) was used to assess the protein expression of six CSC markers (CD133, CD44, ALDH1, SOX2, OCT4, and Nanog) and two EMT markers (E-cadherin and Snail-1). An automated immunostainer (Benchmark Ventana, Tucson, AZ) was used to stain tissue sections following the manufacturer's recommended procedure. The primary antibodies used against the follows: CD133 (1:200; Spring Bioscience, Pleasanton, CA), CD44 (1:200; Thermo Scientific, Fremont, CA), ALDH1 (1:100; BD Biosciences, San Diego, CA), SOX2 (1:100; Cell Signalling, Beverly, MA), OCT4 (1:100; Cell Marque, Rocklin, CA), Nanog (1:100; Epitomics, CA), E-cadherin (1:100; BD Biosciences, San Jose, CA) and Snail-1 (1:500; Santa Cruz Biotechnology, Santa Cruz, CA). IHC results were graded semiquantitatively based on the percentage of cells stained and the intensity of staining [34]. Briefly, the staining intensity was graded as weak (1+), moderate (2+), or strong (3+) and was multiplied by the percentage of positive cells. The total score was then classified as follows: 0-100 = grade 1, 101-200 = grade 2, and 201-300 = grade 3. Grade 2 or 3 tumors were considered to be positive for CSC markers and E-cadherin [35]. For Snail-1, tumors were considered positive when at least 10% of all tumor cells were immunoreactive [36].

### Mutation analyses

Polymerase chain reaction and DNA sequencing with formalin-fixed paraffin-embedded tissue samples were used to analyze *EGFR* mutations in exons 18-21 and *KRAS* mutations at codons 12, 13 and 61, as described previously [37]. Rearrangements of the anaplastic lymphoma kinase gene (*ALK*) were assessed using fluorescence *in-situ* hybridization with an *ALK* probe (Vysis LSI *ALK* Break Apart Rearrangement probe; Abbott Molecular, Park, IL) and a 15% cutoff value, as described previously [38].

### Statistical analyses

The chi-square test, Fisher's exact test and Pearson correlation coefficient were used to evaluate the correlation between CSC marker expression and clinicopathologic parameters. The Kaplan-Meier method, log-rank tests and multivariate Cox proportional hazards regression were used for survival analysis. Two-tailed values of *p* < 0.05 were considered significant. All statistical analyses were performed with SPSS 18.0 (SPSS Inc., Chicago, IL, USA).

## SUPPLEMENTARY FIGURE AND TABLES




