# Supplementary material for: Prognostic significance of stem cell-related marker expression and its correlation with histologic subtypes in lung adenocarcinoma
Source: Oncotarget. 2016 Jun 7;7(27):42502–12. doi: 10.18632/oncotarget.9894 (PMC5173151; doi:10.18632/oncotarget.9894)
Supplement: Supplementary file 1 [file oncotarget-07-42502-s001.pdf]

Prognostic significance of stem cell-related marker expression and its correlation with histologic subtypes in lung adenocarcinoma

SUPPLEMENTARY FIGURE AND TABLES

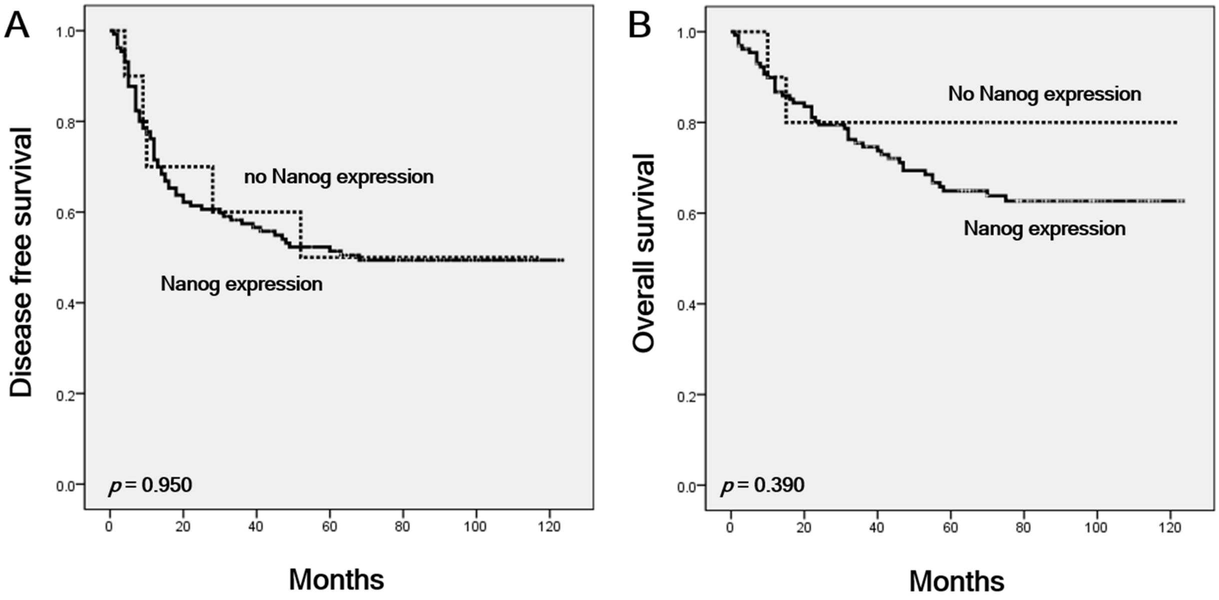

Supplementary Figure S1: Kaplan-Meier curves showing survival among patients with and without Nanog expression in lung squamous cell carcinoma. A. Disease-free survival. **B.** Overall survival. Differences were evaluated using the log-rank test.

Supplementary Table S1: Patient Characteristics

Characteristic	Total (n=368)	ADC (n=226)	SqCC (n=142)	*p*-value
**Sex**				
Male	247 (67.1%)	116 (51.3%)	131 (92.3%)	<0.001
Female	121 (32.9%)	110 (48.7%)	11 (7.7%)	
**Age**				
Median (Range) (years)	63.9 (21–83)	62.6 (21–82)	66.1 (39–83)	
**Smoking history**				
No	144 (39.1%)	134 (59.3%)	10 (7.0%)	<0.001
Yes	224 (60.9%)	92 (40.7%)	132 (93.0%)	
**Tumor size**				
Median (Range) (cm)	3.5 (0.6–16)	3.3 (0.6–16)	3.8 (0.8–9)	
**Pathologic stage**				
I	156 (42.4%)	111 (49.1%)	45 (31.7%)	>0.05
II	95 (25.8%)	36 (15.9%)	59 (41.5%)	
III	105 (28.5%)	70 (31.0%)	35 (24.6%)	
IV	12 (3.3%)	9 (4.0%)	3 (2.1%)	

Abbreviations: ADC, adenocarcinoma; SqCC, squamous cell carcinoma

Supplementary Table S2: Correlations between cancer stem cell marker expression and clinicopathologic characteristics in lung squamous cell carcinoma

See Supplementary File 1
