# Supplementary material for: Prognostic significance of stem cell-related marker expression and its correlation with histologic subtypes in lung adenocarcinoma
Source: Oncotarget. 2016 Jun 7;7(27):42502–12. doi: 10.18632/oncotarget.9894 (PMC5173151; doi:10.18632/oncotarget.9894)
Supplement: Supplementary file 2 [file oncotarget-07-42502-s002.docx]

**Supplementary Table 2. Correlations between cancer stem cell marker expression and clinicopathologic characteristics in lung squamous cell carcinoma**

|  | **Markers, *n* (%)** | | | | | | | | | | | | | | |
| --- | --- | --- | --- | --- | --- | --- | --- | --- | --- | --- | --- | --- | --- | --- | --- |
|  | **CD133** | | | **CD44** | | | **ALDH1** | | | **SOX2** | | | **Nanog** | | |
|  | **Exp** | **No** | ***p*-value** | **Exp** | **No** | ***p*-value** | **Exp** | **No** | ***p*-value** | **Exp** | **No** | ***p*-value** | **Exp** | **No** | ***p*-value** |
| **Tumor size** |  |  |  |  |  |  |  |  |  |  |  |  |  |  |  |
| **≤3 cm** | 0 | 63(44.4) | NA | 54(85.7) | 9(14.3) | >0.05 | 43(68.3) | 20(31.7) | >0.05 | 41(65.1) | 22(34.9) | >0.05 | 59(93.7) | 4(6.3) | >0.05 |
| **>3 cm** | 0 | 79(55.6) |  | 71(89.9) | 8(10.1) |  | 61(77.2) | 18(22.8) |  | 60(75.9) | 19(24.1) |  | 72(91.1) | 7(8.9) |  |
| **Pleural invasion** |  |  |  |  |  |  |  |  |  |  |  |  |  |  |  |
| **Absent** | 0 | 94(66.2) | NA | 83(88.3) | 11(11.7) | >0.05 | 68(72.3) | 26(27.7) | >0.05 | 66(70.2) | 28(29.8) | >0.05 | 88(93.6) | 6(6.4) | >0.05 |
| **Present** | 0 | 48(33.8) |  | 42(87.5) | 6(12.5) |  | 36(75.0) | 12(25.0) |  | 35(72.9) | 13(27.1) |  | 43(89.6) | 5(10.4) |  |
| **Vascular invasion** |  |  |  |  |  |  |  |  |  |  |  |  |  |  |  |
| **Absent** | 0 | 113(79.6) | NA | 100(88.5) | 13(11.5) | >0.05 | 87(77.0) | 26(23.0) | >0.05 | 82(72.6) | 31(27.4) | >0.05 | 105(92.9) | 8(7.1) | >0.05 |
| **Present** | 0 | 29(20.4) |  | 25(86.2) | 4(13.8) |  | 17(58.6) | 12(41.4) |  | 19(65.5) | 10(34.5) |  | 26(89.7) | 3(10.3) |  |
| **Lymphatic invasion** |  |  |  |  |  |  |  |  |  |  |  |  |  |  |  |
| **Absent** | 0 | 81(57.0) | NA | 74(91.4) | 7(8.6) | >0.05 | 62(76.5) | 19(23.5) | >0.05 | 61(75.3) | 20(24.7) | >0.05 | 75(92.6) | 6(7.4) | >0.05 |
| **Present** | 0 | 61(43.0) |  | 51(83.6) | 10(16.4) |  | 42(68.9) | 19(31.1) |  | 40(65.6) | 21(34.4) |  | 56(91.8) | 5(8.2) |  |
| **Pathologic stage** |  |  |  |  |  |  |  |  |  |  |  |  |  |  |  |
| **I** | 0 | 45(31.7) | NA | 39(86.7) | 6(13.3) | >0.05 | 31(68.9) | 14(31.1) | >0.05 | 32(71.1) | 13(28.9) | >0.05 | 42(93.3) | 3(6.7) | >0.05 |
| **II** | 0 | 59(41.5) |  | 52(88.1) | 7(11.9) |  | 46(78.0) | 13(22.0) |  | 42(71.2) | 17(28.8) |  | 52(88.1) | 7(11.9) |  |
| **III** | 0 | 35(24.6) |  | 31(88.6) | 4(11.4) |  | 26(74.3) | 9(25.7) |  | 26(74.3) | 9(25.7) |  | 34(97.1) | 1(2.9) |  |
| **IV** | 0 | 3(2.1) |  | 3(100) | 0 |  | 1(33.3) | 2(66.7) |  | 1(33.3) | 2(66.7) |  | 3(100) | 0 |  |
| **Total** | **0** | **142**  **(100)** |  | **125**  **(88.0)** | **17**  **(12.0)** |  | **104**  **(73.2)** | **38**  **(26.8)** |  | **101**  **(71.1)** | **41**  **(28.9)** |  | **131**  **(92.2)** | **11**  **(7.8)** |  |

Abbreviations: CD, cluster of differentiation; ALDH1, aldehyde dehydrogenase 1; SOX2, sex determining region Y-box 2; Exp, expression

* Statistically significant (*p* < 0.05)
